# A High Gain Embedded Helix and Dielectric Rod Antenna with Low Side Lobe Levels for IoT Applications

**DOI:** 10.3390/s22207760

**Published:** 2022-10-13

**Authors:** Muhammad Nasir, Yulong Xia, Abu Bakar Sharif, Guangjun Guo, Qi Zhu, Masood Ur Rehman, Qammer Hussain Abbasi

**Affiliations:** 1 Department of Electrical Engineering, University of Engineering and Technology, Lahore 39161, Pakistan; 2 Department of Electronic Engineering and Information Science, University of Science and Technology of China (USTC), Hefei 230026, China; 3 Department of Electrical Engineering & Technology, Government College University Faisalabad (GCUF), Faisalabad 38000, Pakistan; 4 James Watt School of Engineering, University of Glasgow, Glasgow G12 8QQ, UK

**Keywords:** Internet of Things (IoT), dielectric rod antenna, aperture, tapered helix, radiation efficiency, side lobes level (SLL), directivity, relative gain, metal reflector, axial ratio (AR), (FBR) front to back ratio

## Abstract

In this paper, a novel embedded helix dielectric rod antenna is presented for high gain radiation with circular polarization (CP) and low side lobe levels for IoT Applications. Different from the conventional dielectric rod antennas, this proposed antenna is an integrated structure that combines the advantages of the helix and dielectric rod antennas. The presented antenna mainly consists of three parts: a tapered helix as primary feeding for CP, a dielectric rod with printed loops embedded for higher directivity, and a dielectric rod end for improving the gain further. After studying and analyzing the working principles of each part, an optimum design operating at 8–9.7 GHz is carried out as an example. A prototype is also fabricated and tested. The measured results show that the prototype can provide 18.41 dB maximum gain within the length of 7.7 *λ*. The side lobe level is below −20 dB, and the axial ratio is better than 1.14 dB in the whole frequency band. Compared with the traditional helix antenna and dielectric rod antenna with the same electric length, the presented antenna has a higher gain with a lower side lobe level and with good polarization purity.

## 1. Introduction

The Internet of Things (IoT) has become a major trend across a wide range of industries, and many IoT-based applications are beginning to scale up from pilot projects. As more IoT use cases are adopted, it is anticipated that the digital transformation of enterprises will accelerate. It is essential to combine the efforts of IoT and 5G technology in order to fully realize the potential of IoT and move toward the goal of pervasive data connectivity [1,2]. The circularly polarized (CP) antennas can suppress the multipath effects and have great flexibility in the orientation of the transmitting and receiving antennas [3]. Thus, CP antennas are widely used in many applications, such as satellite communication and navigation, wireless identification, radar detection, etc. As the requirement for high data throughput grows dramatically, CP antennas with high directivity and wide bandwidth are in great demand to overcome path loss and increase the data rate.

Many types of CP antennas have been designed, such as printed antennas [4,5,6], slot antennas [7], magnetoelectric dipole antennas [8], cavity antennas [9], helix antennas [10], and dielectric rod antennas [11], while the gains of these CP antennas are limited. The gain of these antennas depends on length and diameter [10,11,12]. Typically, a dielectric rod antenna with 11 *λ*–15 *λ* length can obtain a gain of up to 18–20 dBi [11,13].

There are two examples of designing CP dielectric antennas. One is to use linear polarized primary feeding with a rotated tapered dielectric rod [11], and the other is to adopt CP primary feeding with a rotationally symmetric dielectric rod [14,15]. The antenna developed in [9] has good circular polarization performance, but the gain is only 9 dBi within the 4.2 *λ* × 0.5 *λ* aperture. In [16], a bifilar helix is employed as a feeding structure to obtain CP radiation within the reduced dimension, but it can only realize a 9 dBi gain at 8 GHz.

To obtain higher gains, a dielectric rod antenna with a larger size is presented in [17], and a linearly and curvilinear tapered cylindrical dielectric rod fed by a conical waveguide is designed to achieve 22 dBi gain with a total length of 20 *λ*. However, its radiation efficiency will decrease quickly when the length is larger than 7 *λ* [18]. Moreover, the larger length of a typical dielectric rod antenna also increases the side lobe levels. 

In the presented structure, a tapered helix is used as the primary feeding of the dielectric rod antenna for CP. Further, extra tapered printed loops are also embedded with the rod to obtain higher gain and lower side lobe levels. The novelty of the presented structure lies in that, in comparison with previous dielectric rod antennas, extra printed loops are embedded dielectric rods instead of periodic surfaces to increase the gain further. Along with high gain, high polarization purity is successfully achieved for CP radiations.

**Figure 1:**
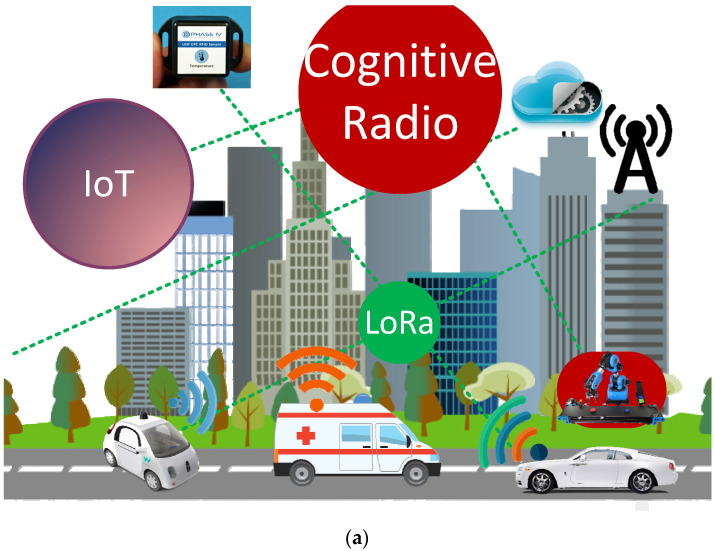

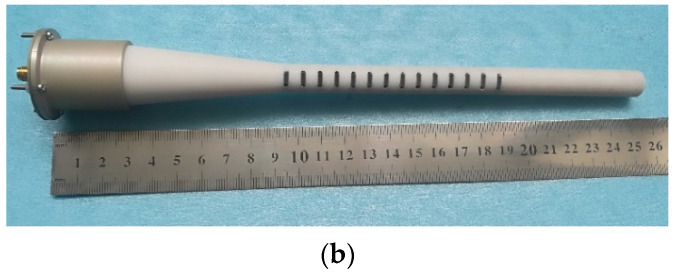
(**a**) Application areas of proposed antenna (**b**) Fabricated prototype of Embedded Helix and dielectric rod antenna.

Moreover, in the proposed embedded structure, double gain can be achieved for the same electrical length of conventional dielectric rod antennas, SLL (Side Lobe Levels) are also lower than in conventional rod antennas, and SLL decreases as antenna length increases, whereas in conventional same class antennas SLL increases with the length of antenna for higher gain. A prototype operating around 9.5 GHz has been designed and fabricated, as shown in Figure 1b. The measured results show that the prototype can provide more gain within the same length of 7.7 *λ*. Additionally, the axial ratio is smaller than 1.14 dB for the entire band. The comparison of simulated and measured results gives a good agreement of theoretical and practical structure.

## 2. Theoretical Analysis

**Figure 2:**
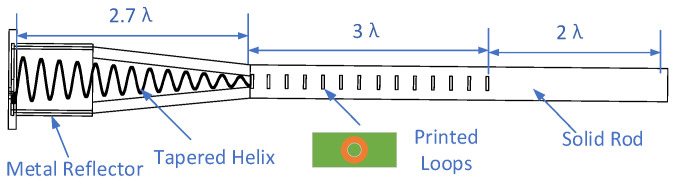
Schematic design of Embedded Helix and dielectric rod antenna.

The configuration of the presented antenna is shown in Figure 2. It is rotationally symmetric and mainly composed of three parts. The first part is a tapered helix inside a tapered dielectric hollow as primary feeding for CP. The second part is a solid dielectric rod embedded with tapered printed loops to improve the directivity. The third part is a solid dielectric rod end to improve the whole radiation pattern.

### 2.1. The First Part

As shown in Figure 2, the first section of this part is a tapered helix inside a uniform hollow dielectric rod covered with 1 *λ* length of the circular metal waveguide. The rest of 1.7 *λ* of the first part is linearly tapered at the same rate as the helix inside. The helix has a uniform turn spacing of 0.21 *λ*, so there are a total of 13 turns for the whole length of 2.7 *λ*. The diameter of the helix turns is linearly tapered from 0.493 *λ* to 0.123 *λ* with the tapering cone angle of α ≈ 7.8°. As the tapered helix being primary feeding, the operating band and directivity can be predicted according to [19]. Additionally, the normalized phase velocity p of wave propagation along the helix can also be calculated based on (1) [19].

(1) $$p=\frac{L_{\lambda }/n}{S_{\lambda }+\left[\left(2n+1\right)/2n\right]}\;$$

where *n* is the turn number of the Helix, *S_λ_* is the normalized spacing between the adjacent turns, and *L_λ_* is the total length of the helix in straighten, and there is *p* = 0.79 for uniform helix. The total length of the tapered helix can be calculated as:

(2) $$L_{\lambda }=\frac{S_{\lambda }tan\frac{\alpha }{2}\;}{2\pi }\left(\frac{t^{2}}{8}+\frac{Q}{2}lnt-\frac{Q^{2}}{8t^{2}}\right)$$

where $t=\sqrt{\varphi_{t}^{2}+Q}+\varphi_{t}$, and $\varphi_{t}=26\pi$, $Q=1+\frac{1}{{tan}^{2}\frac{\alpha }{2}}$. For this tapered helix, there is p≈0.56.

The directivity of helix is calculated as [17]

(3) $$D_{0}\left(dimensionless\right)≅15nC_{\lambda }^{2}S_{\lambda }$$

where $C_{\lambda }$ is the normalized circumference of Helix, and $C_{\lambda }$ can be calculated as follows:

(4) $$C_{\lambda }=\sqrt{{\left(\frac{L_{\lambda }}{n}\right)}^{2}-S_{\lambda }^{2}}\;$$

Thus the theoretical directivity of helix is

(5) $$D_{0}\left(dimensionless\right)≅\left\{\begin{array}{c} 15.8\;\mathrm{dB}\;for\;uniform\;helix\\ 12.6\;\mathrm{dB}\;for\;tapered\;helix \end{array}\right.$$

**Figure 3:**
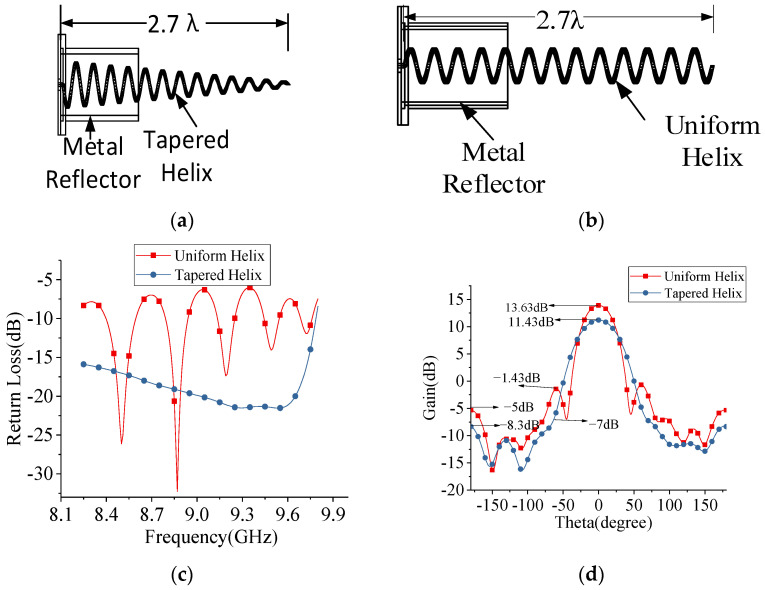
(**a**,**b**) Gives the schematic of tapered helix and uniform helix with metal reflector (**c**) Bandwidth comparison of tapered and uniform helix. (**d**) The gain and side lobe level comparison with tapered and uniform helix.

**Figure 4:**
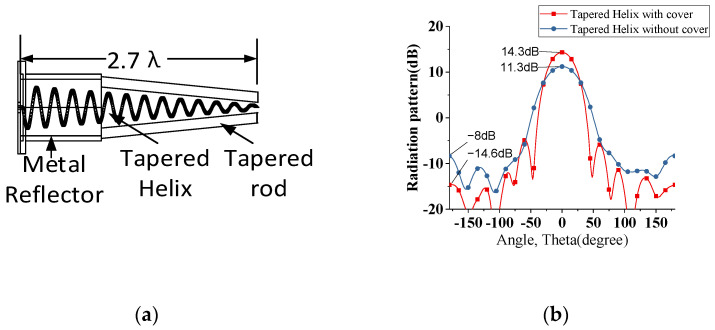
(**a**) Gives the schematic of tapered helix with tapered dielectric cover (**b**) The gain and side lobe level comparison of tapered helix with and without tapered dielectric cover.

According to [20], the bandwidth of a tapered helix is higher than a uniform helix, although a tapered helix has a lower gain than a uniform helix. The simulated results of uniform and tapered helix are shown in Figure 3. Now the key factor is to improve the gain of a tapered helix. The gain of the tapered helix is increased with a tapered dielectric cover of thickness 0.11 *λ*, as shown in Figure 4a,b.

**Figure 5:**
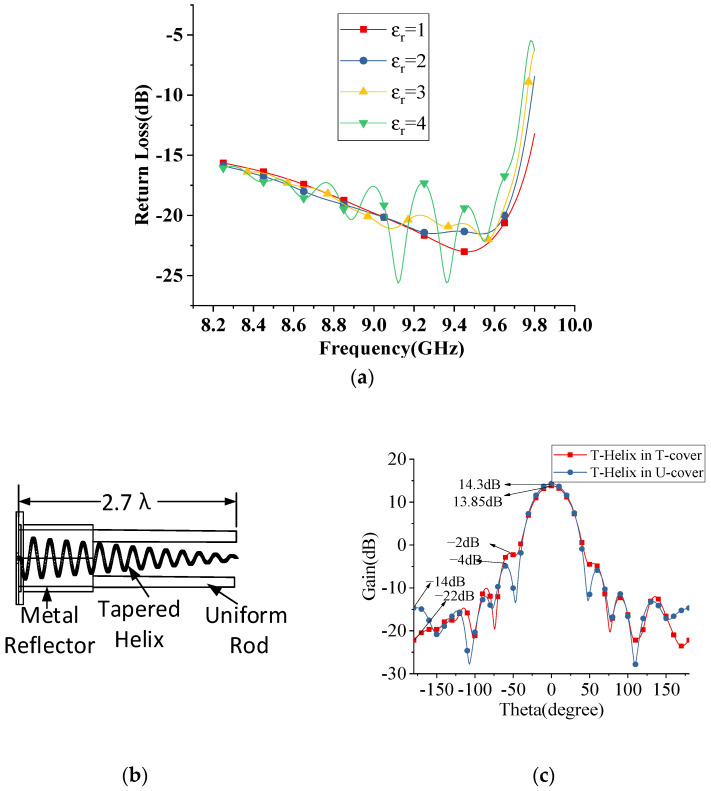
(**a**) Return loss of tapered helix antenna with covering of different dielectric constant (**b**) Schematic of Tapered-helix in Uniform cover (**c**) FBR with and without tapered dielectric cover.

It can be seen from Figure 5a that the dielectric cover has little influence on the return loss of the tapered helix due to the small thickness of the cover [21]. The front-to-back ratio (FBR) of tapered helix with and without tapered dielectric cover is discussed in Figure 5b. The 8 dB front-to-back ratio is improved with a tapered dielectric cover, although little gain effects with this tapering, as shown in Figure 5c. The diameter of the tapering part of the dielectric cover can be selected by using (6) and (7) [22].

(6) $$d_{\max}\approx \frac{\lambda_{0}}{\sqrt{\pi \left(\varepsilon_{r}-1\right)}\;}$$

(7) $$d_{min}\approx \frac{\lambda_{0}}{\sqrt{2.5\pi \left(\varepsilon_{r}-1\right)}}$$

where *d*_max_ and *d_min_* are the maximum and minimum diameters of the dielectric rod supporting the lowest and highest critical frequencies of the operating band, respectively, and *ε_r_* is the relative dielectric constant of the rod.

### 2.2. The Second Part

**Figure 6:**
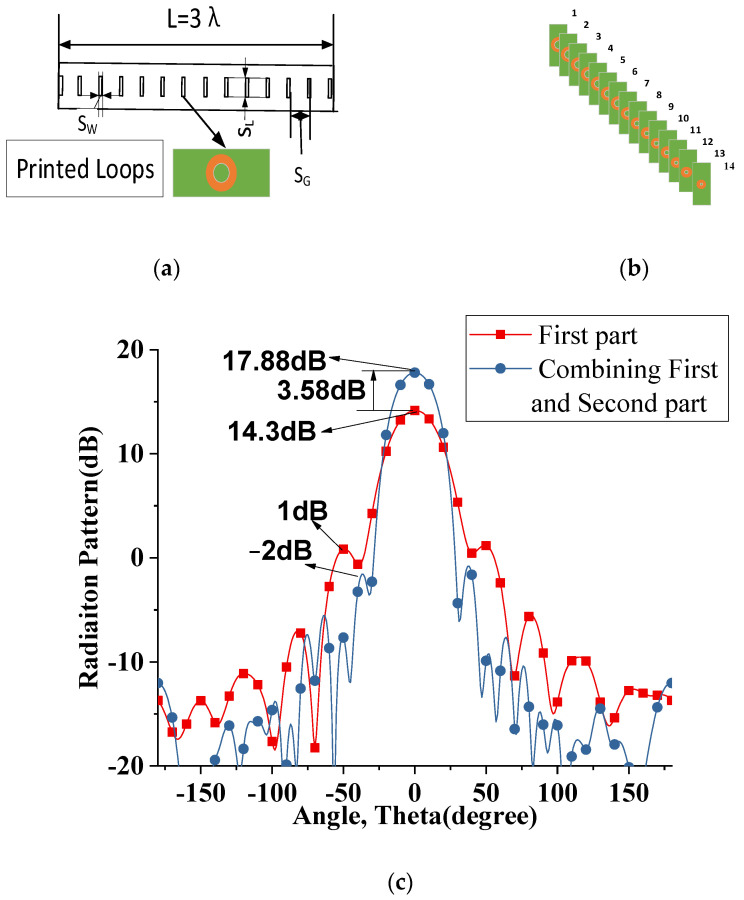

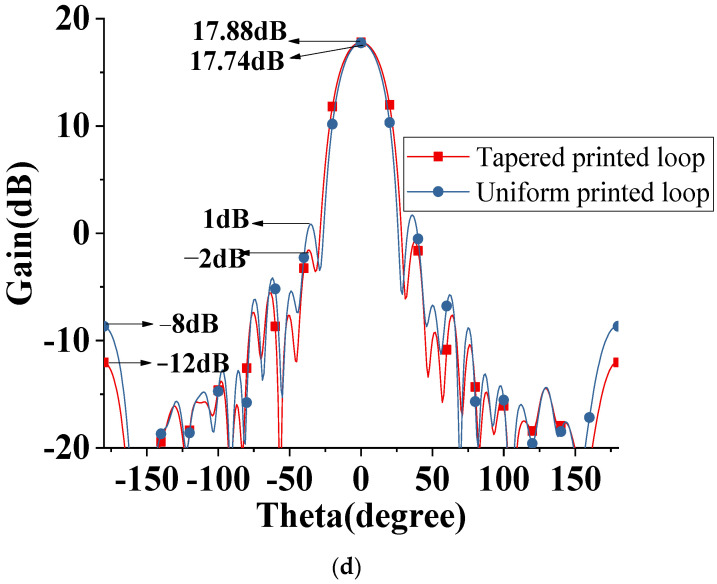
(**a**,**b**) The schematic of embedded printed loops dielectric rod (**c**) Gain and SLL comparison first part and combining first and second part (**d**) Radiation pattern comparison of tapered and uniform printed loop.

As shown in Figure 6a,b, the second part of the presented dielectric rod antenna is a solid dielectric rod embedded with several tapered printed loops. In this design, the spacing between the loops is chosen to be the same as the helix turns of 0.21 *λ*. Thus, for the total length of 3 *λ*, the number of the printed loops is 14. These loops are arranged uniformly along the axis of the dielectric rod with their radius tapered linearly from 2.5 mm to 1.2 mm. These printed loops are fed by the mutual coupling helix in the first part. These embedded printed loops significantly increase the gain and decrease the side lobe level due to the long periodic feeding profile inside the dielectric rod. The side lobe levels are further suppressed by tapering the radius of the printed loops.

To show the effect of the second part on the radiation pattern of the first part, Figure 6c is helpful. It gives a comparison of the radiation patterns between the first part alone and the combination of the first and second parts. It can be seen that this part further improves 3.58 dB gain and a −3 dB side lobe level suppression. Figure 6d shows the radiation pattern comparison of tapered and uniform printed loops. It can be seen that printed tapered loops further suppressed −3dB side lobe levels, and 4 dB FBR is also improved.

### 2.3. The Third Part

**Figure 7:**
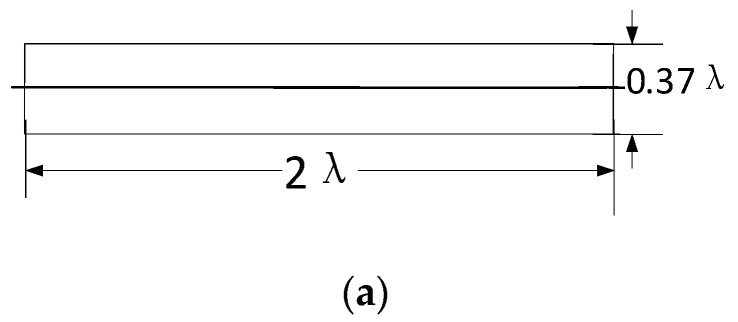

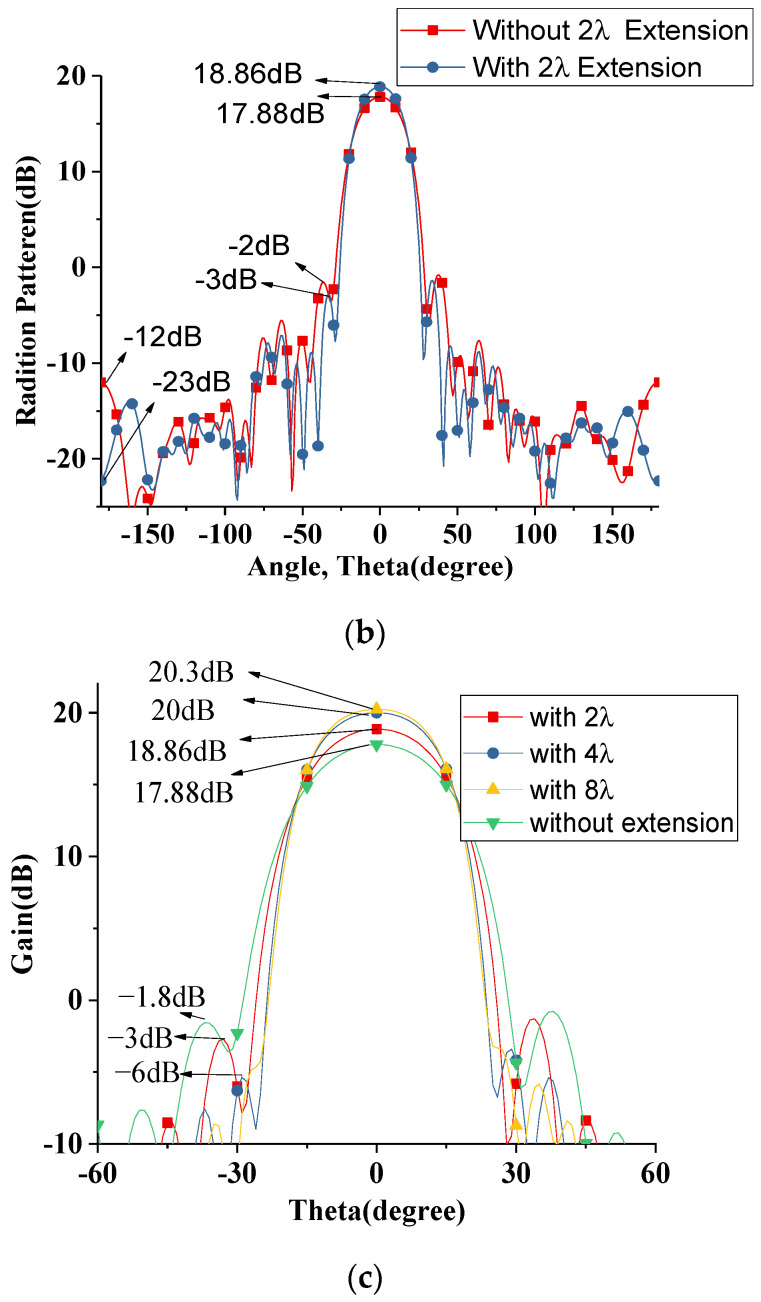
(**a**) Schematic of dielectric rod extension (**b**) Radiation pattern comparison with and without rod extension (**c**) Gain and length of the rod comparison.

The schematic diagram of the third part is shown in Figure 7a. It is a uniform solid dielectric cylinder with a length of 2 *λ* and a diameter of 0.37 *λ*. It is designed to improve the whole radiation pattern; moreover, the length of this part can be adjusted according to the gain requirement, which makes this presented antenna more universal.

Figure 7b provides the radiation patterns comparison of the presented antenna with and without the rod extension. Noticeably, it is seen that this part further adds 1 dB gain along with 11 dB FBR. The relation of the gain with the length of this part is shown in Figure 7c. In increasing lengths of 2 *λ*, 4 *λ*, and 8 *λ*, the gain increases 0.98 dB, 1.14 dB, and 0.3 dB, respectively, and the side lobe levels are suppressed.

## 3. Design and Analysis of the Whole Structure

**Figure 8:**
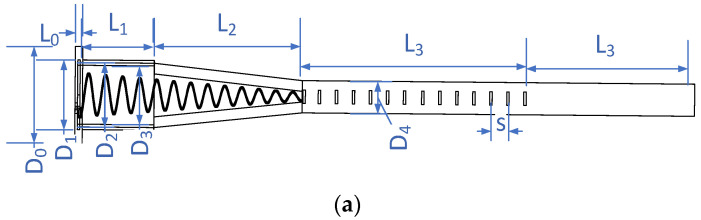

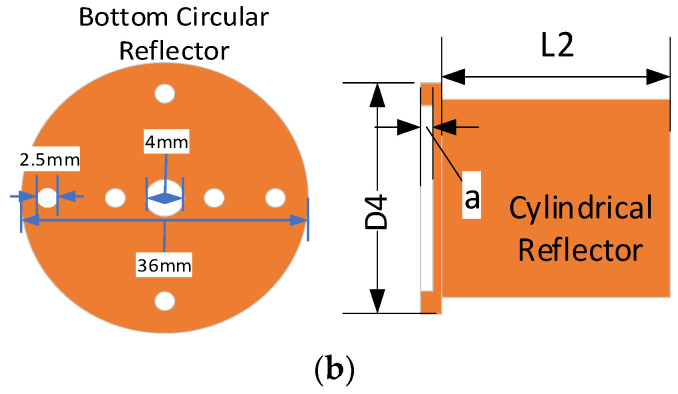
(**a**) Parametric description of the proposed antenna structure (**b**) Layout of metal reflector.

By integrating the three parts presented above, the whole structure of the presented dielectric rod antenna is shown in Figure 8a. As the primary feeding, the tapered helix of wire 0.7 mm diameter is left-hand circularly polarized (LHCP) and is designed to operate at 8–9.7 GHz. The helix is planted on a circular bottom reflector and embedded inside the dielectric hollow covered by a cylindrical metal waveguide. The reflector dimension parameters are shown in Figure 8b.

In the presented antenna, the printed loops are designed on the substrate with a thickness of 2 mm and a relative dielectric constant of 2.65. The dielectric structure is designed with Teflon material. All the design parameters are given in Table 1.

According to Table 2, the antenna developed in [11] has good circular polarization performance, but the gain is only 9 dBi within the 4.2 *λ* × 0.5 *λ* aperture. According to [12], the gain is 17.5 dB can be achieved with a length of 11.9 *λ* and side lobe level are on −10 dB. The side lobe levels are better in [13,14], but the gain is low as compared to the proposed antenna. In [16], a Compact High Gain Dielectric Rod Antenna Array is designed to get high gain with low side lobe levels, but the gain for a single element is only 9 dB which is very low. It is concluded that the performance of the proposed antenna is better than all conventional dielectric rod antennas.

**Figure 9:**
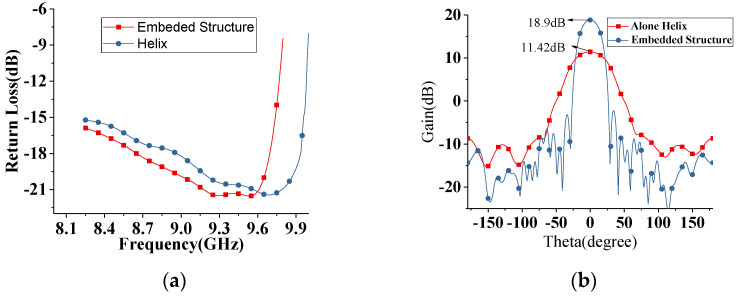
(**a**) The return loss of Tapered helix with metal reflector and whole structure (**b**) the gain comparison of Helix and whole structure.

Figure 9a shows a comparison of the simulated return loss between the helix alone and the whole structure. It can be seen that the return losses are better than −15 dB in the working band of 8–9.7 GHz. Additionally, these two return losses are almost the same, which means that the operating band of the presented antenna is mainly determined by the primary feeding. Figure 9b provides the radiation patterns at 9.5 GHz, where it is found that the gain of the embedded structure is 7.43 dB more than the gain of a single helix and the side lobe level is also very low for the proposed design.

**Figure 10:**
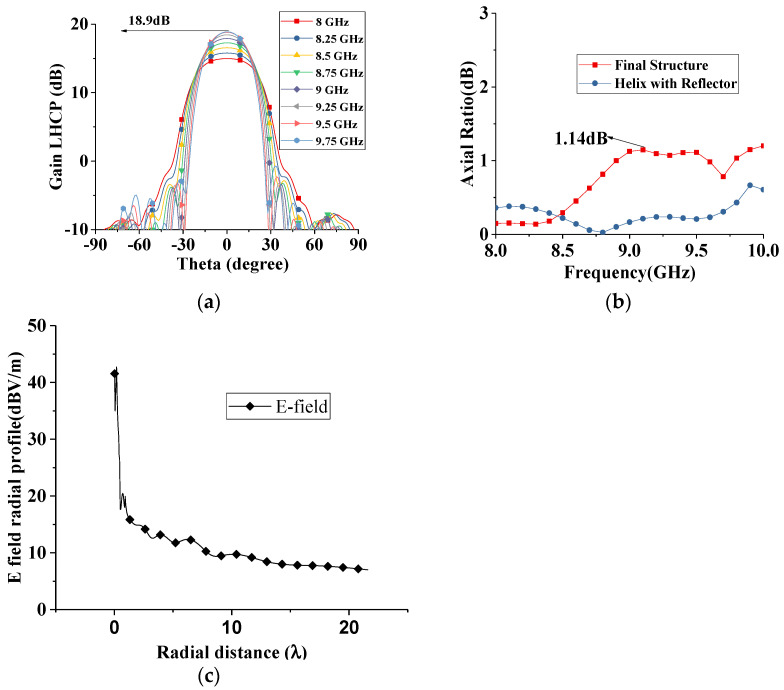
(**a**) Radiation patterns of the whole structure for entire working band (**b**) Axial ratio for helix and final structure in entire operational band (**c**) Electric field radial profile of presented antenna.

Figure 10a,b shows the radiation patterns and axial ratio at the zenith of the presented antenna for the whole working band. It can be found that the gain of the antenna is higher than 15 dB in the whole band, and a maximum gain of 18.9 dB is achieved at 9.7 GHz. The side lobe levels are below −20 dB in the whole band. Additionally, good polarization purity can be found; in Figure 10b, the axial ratio is below 1.14 dB in the whole band. The mathematical description of the amplitude of the field along the surface wave antenna is described in [12], and the electric field distribution of the proposed structure is shown in Figure 10c.

## 4. Fabrication and Measurement

**Figure 11:**
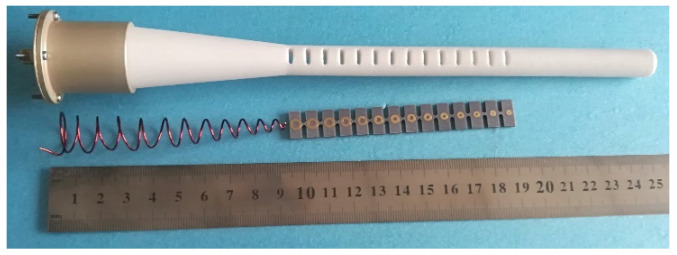
Fabricated parts of Embedded Helix and dielectric rod antenna.

A prototype of the presented dielectric rod antenna was fabricated to verify the theoretical analysis, as shown in Figure 11. In the prototype, the helix was made of copper wire and placed into a Teflon hollow with a relative dielectric constant of 2.1. Then, the dielectric rod was fixed with aluminum reflectors. A 50 Ω SMA connector is used as the feed port, and the inner and outer conductors connect with the helix and the bottom reflector, respectively. The printed loops were fabricated on the F4B substrate with a thickness of 2 mm and a relative dielectric constant of 2.65. Finally, these printed loops were separated and inserted into the slots on the dielectric rod in series to construct the compact structure.

**Figure 12:**
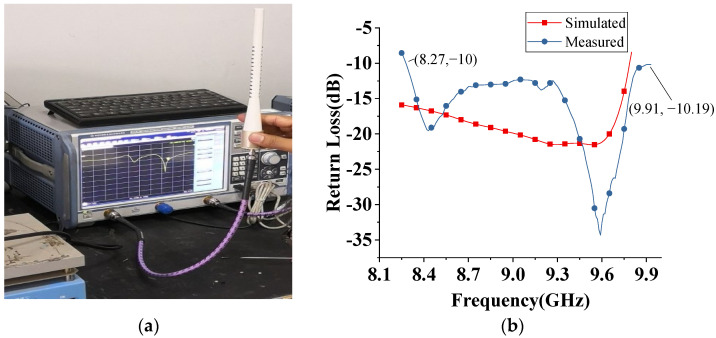
(**a**) Return Loss measurement setup (**b**) Simulated and measured return loss of Embedded Helix and DR antenna.

Figure 12a shows the return loss measurement of the prototype. It is seen from Figure 12b that the measured return loss is almost below −12 dB in the whole band. There still exists some difference between the simulated and measured results. This is mainly due to the fabrication errors of the helix, especially the deviation of the impedance-matching segment on the first turn.

**Figure 13:**
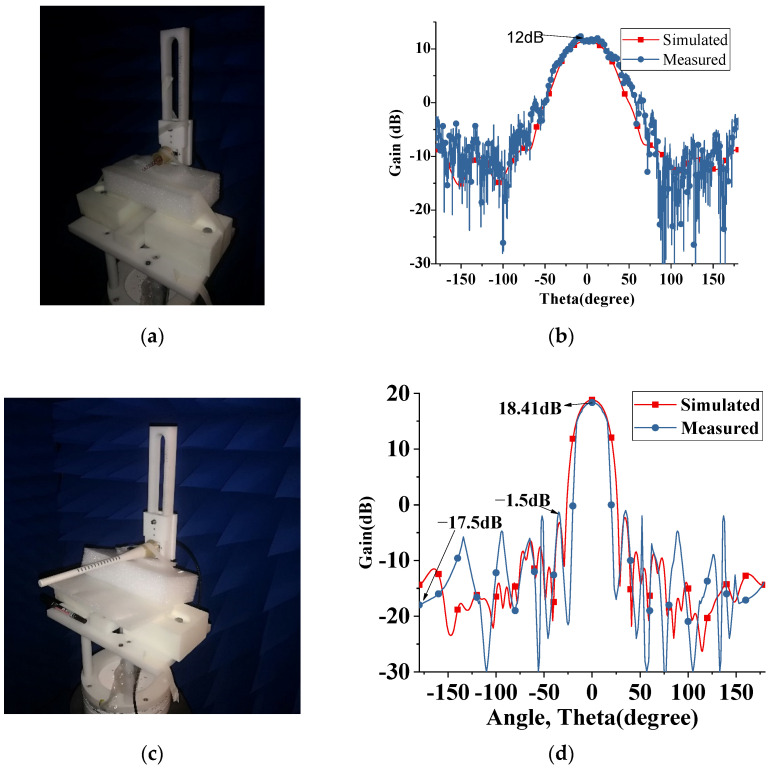
(**a**) Radiation patterns Measurement setup of Helix (**b**) simulated and measured radiation pattern comparison of Helix alone (**c**) radiation pattern measurement of the proposed structure (**d**) simulated and measured radiation pattern comparison of the proposed structure.

To verify the theoretical analysis better, the single helix, as well as the whole structure, are both measured to compare with the simulation, as shown in Figure 13. Figure 13b shows that the measured and simulated radiation patterns of the alone helix are almost the same. Similarly, in Figure 13d, it is found that the measured radiation patterns of the proposed embedded structure helix meet well with the simulated results. The small difference on the side lobe level is mainly caused by fabrication errors and environmental noise in the measurement. Additionally, the measured gain of the whole structure is about 0.4 dB lower than that of the simulated. This is mainly due to the losses caused by the solder, SMA connector, and dielectric. Further, Teflon material is used for the proposed antenna structure where Teflon dielectric constant varies from 2 to 2.04 for temperatures 10 °C to 50 °C, respectively [23]. Furthermore, it is already discussed that the dielectric constant has little influence on return loss in the proposed structure, as mentioned in Figure 4a. It is concluded that temperature has a negligible effect on the proposed antenna’s performance.

## 5. Conclusions

In this paper, a novel embedded helix dielectric rod antenna is presented for high gain radiation with CP and low side lobe level. Different from the conventional dielectric rod antenna, the innovative design consisting of three main parts contributes to gain enhancement and SLL suppression significantly. The gain of 2.7 *λ* long tapered helix with tapered dielectric cover gives the improvement of 3 dBi for the same length. The 3 *λ* printed loops embedded with a solid dielectric rod are introduced to improve the directive gain of the structure 6.58 dB further. A 2 *λ* long uniform solid dielectric rod adds almost 2 dB directive gain; this part also provides design feasibility for the required gain. Additionally, the presented antenna has a simple structure, compact size, low cost, and highest radiation efficiency. It is useful for satellite communication, commercial wireless LAN with high resolution, and IoT Applications.

## Figures and Tables

**Table 1:** Dimension parameters of the proposed structure.

Symbol	Value (mm)	Symbol	Value (mm)
**L_0_**	2	D_3_	22
**L_1_**	30	D_2_	24
**L_2_**	57.48	D_1_	26
**L_3_**	97.2	D_4_	12
**L_4_**	64	S	6.9
**a**	1	D_0_	36
**S_W_**	2	S_L_	5
**S_G_**	6.9		

**Table 2:** Comparison Table.

Reference	[11]	[12]	[13]	[14]	[16]	This Work
**Frequency** **(GHz)**	33	10	5.5	5.6	8	9.25
**Gain** **(dB)**	9	17.5	15	16.7	9	18.41
**SLL (dB)**	−10	−10	−20	−15	−7	−20
**Dimension**	10 *λ* × 0.75 *λ*	11.9 *λ* × 0.6 *λ*	6 *λ* × 0.8 *λ*	3.2 *λ* × 1.2 *λ*	1 *λ* × 0.5 *λ*	7.7 *λ* × 0.74 *λ*
**Polarization**	Circular	Linear	Linear	Linear	Linear	Circular

## Data Availability

Not applicable.
